# LN Monocytes Limit DC-Poly I:C Induced Cytotoxic T Cell Response *via* IL-10 and Induction of Suppressor CD4 T Cells

**DOI:** 10.3389/fimmu.2021.763379

**Published:** 2021-10-06

**Authors:** Anita Tewari, Miglena G. Prabagar, Sophie L. Gibbings, Kavita Rawat, Claudia V. Jakubzick

**Affiliations:** ^1^ Department of Microbiology and Immunology, Geisel School of Medicine, Hanover, NH, United States; ^2^ Department of Pediatrics, National Jewish Health, Denver, CO, United States

**Keywords:** dendritic cells, Ly6C^+^ monocytes, antigen-presentation, IL-10, cytotoxic T cells, Poly I:C, suppressor CD4^+^ T cells, double-stranded RNA

## Abstract

Every immune response has accelerators and brakes. Depending on the pathogen or injury, monocytes can play either role, promoting or resolving immunity. Poly I:C, a potent TLR3 ligand, licenses cross-presenting dendritic cells (DC1) to accelerate a robust cytotoxic T cells response against a foreign antigen. Poly I:C thus has promise as an adjuvant in cancer immunotherapy and viral subunit vaccines. Like DC1s, monocytes are also abundant in the LNs. They may act as either immune accelerators or brakes, depending on the inflammatory mediator they encounter. However, little is known about their contribution to adaptive immunity in the context of antigen and Poly I:C. Using monocyte-deficient and chimeric mice, we demonstrate that LN monocytes indirectly dampen a Poly I:C induced antigen-specific cytotoxic T cell response, exerting a “braking” function. This effect is mediated by IL-10 production and induction of suppressor CD4^+^ T cells. In a metastatic melanoma model, we show that a triple-combination prophylactic treatment consisting of anti-IL-10, tumor peptides and Poly I:C works because removing IL-10 counteracts the monocytic brake, resulting in significantly fewer tumors compared to mice treated with tumor peptides and Poly I:C alone. Finally, in human LN tissue, we observed that monocytes (unlike DCs) express high levels of IL-10, suggesting that anti-IL-10 may be an important addition to treatments. Overall, our data demonstrates that LN monocytes regulate the induction of a robust DC1-mediated immune response. Neutralization of either IL-10 or monocytes can augment Poly I:C-based treatments and enhance T cell cytotoxicity.

## Introduction

The mononuclear phagocyte (MP) system in the healthy lung is comprised of tissue-specific alveolar macrophages, interstitial macrophages, dendritic cells (DCs) and monocytes (1). Of these MPs, DCs and monocytes can acquire antigen in the lung, migrate to the lung-draining lymph node (LLN), and present antigen to induce T cell differentiation. The induction of naïve T cells into effector T cells is mainly attributed to two DC subtypes, DC1 and DC2, with further subdivision of DC2 into subsets (2–5). In a previous publication, we demonstrated that DC1 is the main driver of a Poly I:C-induced CTL response (6). When lung was stimulated with Poly I:C and antigen, DC1 was shown to be essential for differentiating cytotoxic T cells (CTL) (6). DC2 could cross-present the antigen and induce T cell proliferation but could not differentiate the antigen-specific T cells to become effector T cells. In addition to DCs, the LN contains monocytes, which are as abundant as any DC subset (1, 7–9), so in this study we ask, what role are the LN monocytes playing.

Monocytes represent ~5-10% of circulating PBMCs and have a half-life of ~20 hours (7, 10). They are continuously trafficking into tissue and LNs and are often referred to as inflammatory monocytes or macrophages. This is a misnomer, as Ly6C^+^ monocytes continuously enter tissue and LNs in healthy animals (during steady state) to survey the environment (7). The rapid migration into tissue is illustrated in parabiotic mice where Ly6C^+^CD62L^+^ monocytes never reach 50:50 equilibrium regardless of the amount of time the mice share circulation (7, 11–13). Most importantly, the trafficking of monocytes, like DCs, into tissue and LNs does not require commensals (7), suggesting that monocytes are critical players in immune regulation both in steady state and inflammation. Based on the turnover, number and surveying property of monocytes, many questions arise for the role of monocytes; particularly after it has been shown that monocytes are not readily replacing steady state macrophages in some cases, such as alveolar macrophages and microglia, while in others this replacement occurs regularly such as intestinal and liver macrophages (7, 11, 14–22).

Once monocytes enter tissue, they can display phagocytic properties and secrete inflammatory mediators such as TNF and NO, or anti-inflammatory mediators such as IL-10 and VEGF (8, 17, 23, 24). Although these examples suggest that monocytes can have macrophage-like properties, monocytes can also display DC-like properties, upregulating CCR7 and migrating down afferent lymphatics from tissue to LNs (7, 25). Upon entering the LNs, monocytes present exogenous antigen to cognate T cells and induce their differentiation into effector CD4 or CD8 T cell (i.e., Th1, Th2, Th17, T reg, CTL etc.) (9, 19, 26–32). All in all, the stimulus (i.e., pathogen associated molecular pattern) and environment (i.e., growth factors) dictate the outcome and role of monocytes during an immune response, highlighting their plasticity.

The importance of understanding how LN monocytes contribute to the adaptive immune response is largely based on the growing number of clinical trials that use DC-based vaccine designs containing tumor-associated antigens or personalized neoantigens with Poly I:C (33, 34). The efficacy of such vaccines was demonstrated in recent studies in which treatment of stage III melanoma patients with personalized neoantigens and Poly I:C resulted in complete tumor regression, with no recurrence at 2-year follow-up (33, 34). Since monocytes contribute to the development of effector T cells or suppressor T cells and are highly present in the draining LNs, we set out to address the role of monocytes in the context of antigen and Poly I:C vaccination, and perhaps provide insight on how to enhance the efficacy of this promising cancer and viral vaccine.

## Materials and Methods

### Mice

Six to eight-week-old C57BL/6 Ly5.1 (CD45.1), Ly5.2 (CD45.2), WT mice were purchased from Charles River or Jackson Laboratory. CCR2^-/-^, IL-10^-/-^, H–2K^bm1^, OTI, OTII, MHCII^-/-^ and IL-10 GFP (Tiger) mice were purchased from Jackson Laboratory. PDL1/2^-/-^ mice were kindly provided by Dr. Daniel Barber and Keith Kauffman. OTII mice were crossed with IL-10 GFP to generate OTIl IL-10 GFP reporter mice. All mice were genotyped upon arrival and prior to their use in experiments. Mice were housed in a specific pathogen-free environment at National Jewish Health and Dartmouth Hitchcock Medical College, an AAALAC accredited institution, and used in accordance with protocols approved by the Institutional Animal Care and Utilization Committee.

### Bone Marrow Chimera Mice

Eight-week-old CD45.1 or CD45.2 C57BL/6 mice were lethally irradiated with 900 rads and reconstituted intravenously at a bone marrow (BM) cell ratio of 80:20 from CCR2^-/-^ and either WT, H–2K^bm1^, PDL1/2^-/-^, or IL-10^-/-^ mice. Six to eight weeks post-reconstitution, mice were assessed for chimerism prior to use.

### Flow Cytometry and LN Digestion

Lung-draining LNs were teased with 26G needles and digested in 1ml of 2.5 mg ml^−1^ collagenase D (Roche) solution in 1X RPMI at 37°C for 30 min. After incubation, 100 μl of 100 mM EDTA was added to stop the enzymatic reaction. The cells were pipetted up and down with glass Pasteur pipets and then passed through a 70 μm nylon filter. Single cell suspensions were collected and centrifuged at 300 g for 5 min. Cells were stained with the following monoclonal Abs: Phycoerythrin (PE)-conjugated to Vα2, CD4 and CD64; PerCP-Cy5.5- conjugated to CD11b and CD4; PE-Cy7-conjugated to CD11c and CD44; BUV395-conjugated to CD11b; BUV805-conjugated to CD8a, fluorescein isothiocyanate (FITC)-conjugated to CD4, CD45.1 and CD103; allophycocyanin (APC)-conjugated to Vβ51, CD45.2, CD45 and CD103; APC-Cy7-conjugated to CD45.2 and MHCII; and BV510 conjugated to Ly6C and MHCII. The PE-conjugated MHC Tetramer H-2kb OVA SIINFEKL was purchased from MBL international. The viability dye 4’,6-diamidino-2-phenylindole (DAPI) (#D9542, Sigma) was added immediately before each sample acquisition on a BD Symphony A3 analyzer (BD Biosciences). Data were analyzed using FlowJo (Tree Star, Ashland, OR). Antigen-specific antibodies and isotype controls were obtained from BioLegend, eBioscience and BD Biosciences.

### Proliferation of OVA-Specific Transgenic OTI or OTII T Cells

Spleen cells from OTI or OTII GFP mice, in which the TCR of CD8^+^ or CD4^+^ T cells are restricted to an OVA peptide, were labeled with 10 μM carboxyfluorescein succinimidyl ester (CFSE) for 10 mins. Half a million of these cells were transferred intravenously into recipient mice 1 day before intranasal delivery of 2 μg soluble OVA and 20 μg Poly I:C. Mice were sacrificed 3 days after intranasal deliveries for analysis of T cell proliferation (CFSE dye dilution) in the LLN. For OTI: Isolated cells were cultured 5 hours in RPMI 10% FCS containing 10 μM OVA peptide (257-264) and Brefeldin A 10 μg/ml. Following surface staining, BD Foxp3 intracellular staining kit was used. Cells were stained with APC-conjugated to IFNγ, PerCP-Cy5.5-TNFα, or isotype controls (BD Pharmingen). For OTII: Isolated cells were cultured 5 hours in RPMI 10% FCS containing 10 μM OVA peptide (323-339) and Brefeldin A 10 μg/ml. Following stimulation, cells were stained with PE-conjugated to CD4, APC-conjugated to Vβ51, PE-Cy7-conjugated to Vα2, APC-Cy7-conjugated to CD44, BUV805-conjugated to CD8 (BD Pharmingen, eBioscience or Biolegend).

### Quantitative Reverse-Transcription PCR

Monocytes from CD11b enriched cell suspension (Miltenyi) were sorted (FACSAriaIII sorter). Total RNA was extracted using the RNeasy Minikit (Qiagen). Equal amounts of total RNA from each sample were reverse transcribed into cDNA using SuperScript™ VILO™ Master Mix (Invitrogen). qPCR was set up using PowerUp SYBR Green PCR Master Mix (Applied Biosystems) and analyzed on Bio-Rad C-1000 CFX96. RT-PCR primers for IL-10: Forward-GGTTGCCAAGCCTTATCGGA; Reverse-ACCTGCTCCACTGCCTTGCT. Relative quantification of gene expression was performed by normalizing expression levels of genes of interest over housekeeping (Thoc1) gene within each sample followed by relative expression to control (mean of triplicate).

### B16F10 Lung Melanoma Model

B16F10 melanoma cells were purchased from ATCC (CRL-6475) and maintained in RPMI with 10% FCS, 1% Pen/Strep/L-glutamine (Sigma), 1% non-essential amino acids (Sigma), 1% sodium pyruvate (Sigma), 10 mM HEPES (Sigma) and 0.1 mM β-mercaptoethanol.

#### Immunization With B16F10 Peptides, Poly I:C, and Anti-IL-10

Melanoma vaccine was freshly prepared prior to inoculation. Vaccine consisted of 10 μg each peptide premelanosome protein (Pmel17 or gp100) (25-30; seq: EGSRNQDWL, Anaspec) and tyrosinase-related protein-2 (TRP-2) (180-188; seq: SVYDFFVWL, Anaspec), and 50 μg Poly I:C (TLR3 agonists). To deplete IL-10, some mice were given three i.p. injections of anti-IL-10 (JES5-2A5 clone, BioXcell) at days 2 (300μg), 5 (100μg) and 22 (100μg).

#### Tumor Assessment Post Prophylactic Therapy

After treatment, mice were intravenously challenged with 2 × 10^5^ viable B16F10 cells and euthanized 16 days post injection. Lungs of mice were perfused with PBS and then inflated with 1% agarose. Experimentally blind laboratory member counted the B16F10 lung surface metastases.

### Human Lung-Draining LNs

We received de-identified non-diseased human lung with attached LLNs from the International Institute for the Advancement of Medicine (Edison, NJ, USA). We selected donors without a history of chronic lung disease and with reasonable lung function with a P_a_O_2_/F_i_O_2_ ratio of >225, a clinical history and X-ray that did not indicate infection, and limited time on a ventilator. Lungs were removed en bloc in the operating room and included the trachea, LNs and pulmonary vessels. Pulmonary arteries were perfused in the operating room with cold histidine-tryptophan-ketoglutarate (HTK) solution to preserve endothelial cell function and prevent intravascular clot formation. The lungs were submerged in HTK, and immediately shipped on ice. All lungs were processed within 24 hours of removal. The lungs were visually inspected for lesions or masses and were eliminated from the study if grossly abnormal. Peribronchial LNs were identified and removed. Procedure additionally described in (35, 36). The Committee for the Protection of Human Subjects at National Jewish Health approved this research. RNA sequencing was completed as previously described (11).

### Statistics

Statistical analysis was conducted using Prism software. All bar graphs are expressed as the mean ± SEM. Statistical tests were performed using two-tailed Student’s t-test and ANOVA. P < 0.05 was considered statistically significant.

## Results

### The Absence of LN Monocytes Significantly Increases Poly I:C Induced Antigen-Specific CTL Response

**Figure 1:**
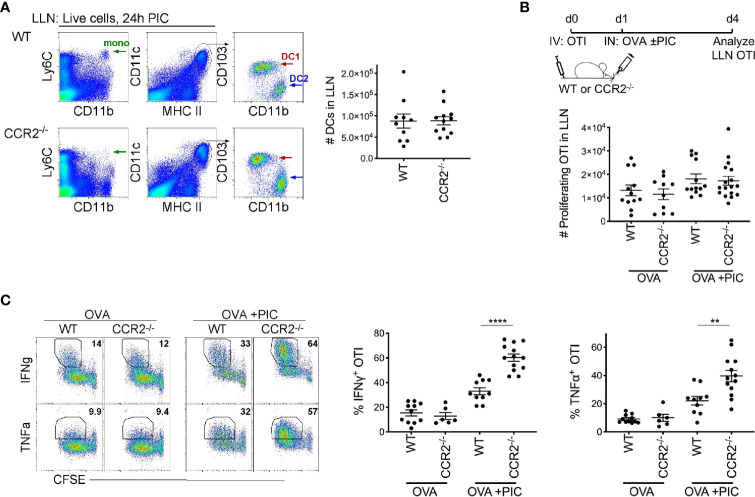
Lymph node monocytes suppress Poly IC-induced antigen-specific cytotoxic CD8^+^ T cells. **(A)** Flow plots of LLN from WT and CCR2^-/-^ mice were isolated 24 hours postimmunization with OVA and Poly IC. Gated live cells illustrate Ly6C^+^CD11b^+^ monocytes (left plots) and CD11c^+^MHCII^high^ population (middle plots), CD103^+^ DC1 and CD11b^+^ DC2 (right plots). Scatter plot, each dot represents a mouse. Total number of migratory DCs in the LLN 24 hours post immunization. Data are 2 independent experiments. **(B)** Immunization protocol for CTL response, top. Total number of proliferating OTI T cells 3 days post- immunization. Data are cumulative from 3 independent experiments with n = 4 per group. **(C)** Representative flow plots display gating strategy of cytokine production from WT and CCR2^-/-^ mice, left. Scatter plot analysis of cytokine frequency from individual mice, right. Data are cumulative from 2-3 independent experiments of n = 3,4 per group. Data are shown as mean ± SEM. Each dot represents a mouse. ****P < 0.0001. **P value < 0.01.

Monocytes require CCR2 to exit the bone marrow and enter tissue under steady-state and inflammatory conditions (7, 37). As anticipated, 24 hours post intranasal delivery of antigen and Poly I:C, the total number of migratory DCs in the LLN was similar between WT and CCR2^-/-^ mice (**Figure 1A**). In contrast, monocytes are substantially deficient in the CCR2^-/-^ mice compared to WT mice (17, 23, 37).

To examine the role of LN monocytes in a Poly I:C-induced antigen-specific CTL response, CFSE labeled naïve OVA-specific CD8^+^ T cells (OTI) were adoptively transferred into WT and CCR2^-/-^ mice. Three days post inoculation of antigen and Poly I:C, LLN OTI T cells were analyzed for the production of CTL cytokines, IFNγ and TNFα. Although we observed a relatively similar number of proliferating CFSE^+^ OTI T cells between WT and CCR2^-/-^ mice (**Figure 1B**), the frequency of IFNγ and TNFα producing OTI T cell was significantly greater in CCR2^-/-^ mice compared to WT mice (**Figure 1C**), showing that monocytes downregulate the intensity of cytokine production in proliferated OTI T cells.

### Temporary Depletion of LN Monocytes Significantly Increases Poly I:C Induced Antigen-Specific CTL Response

To support the findings in the CCR2^-/-^ mice, where LN monocytes are continually deficient, we examined whether temporary depletion of monocytes would also result in an increased Poly I:C induced CTL response. Using anti-Gr1, monocytes were temporarily depleted in WT mice on the same day as the inoculation of antigen and Poly I:C. A caveat to note is that in addition to monocytes, anti-Gr1 depletes neutrophils, plasmacytoid DCs and Ly6C^+^ T cells but not DCs for up to 2-3 days (6, 38). As observed in CCR2^-/-^ mice, WT mice with transiently depleted monocytes displayed a significant increase in IFNγ and TNFα producing antigen-specific CD8^+^ T cells compared to mice treated with control antibody (**Supplementary Figure 1**). Collectively, data from mice either constitutively (CCR2^-/-^) or temporarily (*via* anti-Gr1 treatment) depleted of LN monocytes suggest that the role of monocytes is to restrict an exaggerated CTL response in the context of antigen and Poly I:C.

### IL-10 Production by LN Monocytes Is Required to Dampen an Enhanced CTL Response

**Figure 2:**
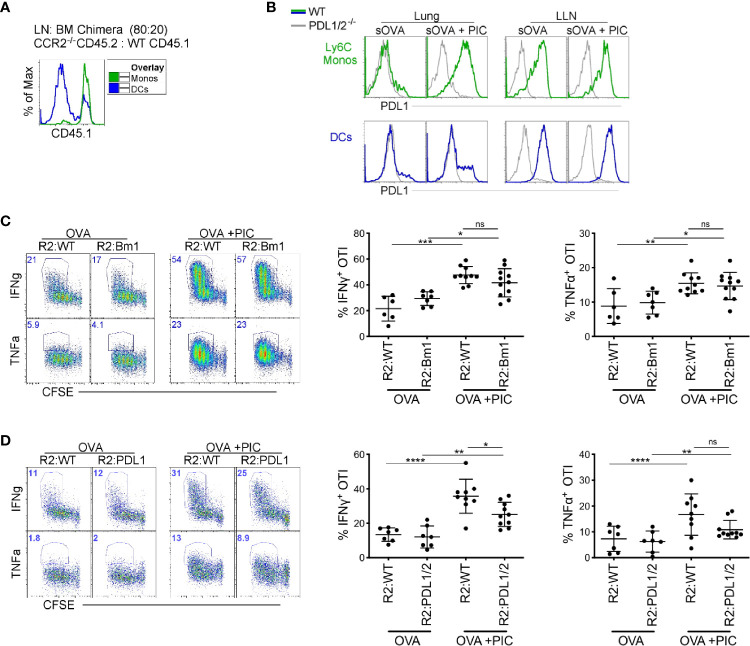
Direct antigen presentation and PDL1 expression by monocytes are dispensable for suppression of cytokine production in antigen-specific CD8^+^ T cells **(A)** Lethally irradiated mice were reconstituted 80:20 with CCR2^-/-^ CD45.2 or WT CD45.1 BM cells. Six weeks post-reconstitution, LNs were assessed for monocyte BM derivation. Flow plots illustrate monocyte derivation was ~100% derived from CCR2 sufficient, WT CD45.1 BM cells compared to DC subtypes which were mainly derived from CCR2^-/-^ CD45.2 BM with a portion from WT CD45.1 BM. **(B)** WT and PDL1/2^-/-^ mice were treated with OVA or OVA and Poly IC, 24 h post treatment, PDL1 protein expression was examined on lung and LN monocytes (top) and total migratory DCs (bottom). **(C)** Lethally irradiated mice were reconstituted 80:20 with CCR2^-/-^ and H–2K^bm1^ BM cells: R2:Bm1 chimeric mice. Control mice were reconstituted 80:20 with CCR2^-/-^ and WT BM cells: R2:WT chimeric mice. Six-eight weeks after reconstitution, mice were immunized as described in **Figure 1B** and cytokine production was examined in OTI proliferating T cells. Right panels represent flow plots demonstrate gating strategy for cytokine production by proliferating OTI T cells. Left, scatter plot analysis of cytokine frequency from R2:WT and R2:Bm1 chimeric mice. Data are 3-4 independent experiments n=3-5 mice per group. Each dot represents a mouse. **(D)** Lethally irradiated mice were reconstituted 80:20 with CCR2^-/-^ and PDL1/2^-/-^ BM cells: R2:PDL1/2 chimeric mice. Control mice were reconstituted 80:20 with CCR2^-/-^ and WT BM cells: R2:WT chimeric mice. Scatter plot analysis of cytokine frequency from R2:WT and R2:PDL1/2 chimeric mice. Data are 2 independent experiments, n=3-5 mice per group. Data are shown as mean ± SEM. Each dot represents a mouse. ****P <0.0001. ***P value < 0.001. **P value < 0.01. *P value < 0.05. Non-significant (ns).

We investigated three possible mechanisms by which monocytes could dampen a Poly I:C induced CTL response. First, we hypothesized that monocytes directly interfere with the cross-presentation and cross-priming of CTLs by TLR3 stimulated DC1s. To address this hypothesis, we took advantage of the H–2K^bm1^ mice which have mutations to the antigen binding site of MHCI, rendering antigen presenting cells incapable of inducing proliferation of OTI T cells (39). Lethally irradiated CD45.1 C57BL/6 mice were reconstituted with a mixture of 80% CCR2^-/-^ and 20% H–2K^bm1^ bone marrow (BM) cells or with 20% CD45.1 WT BM in control mice. In this competitive setting, CCR2-sufficient BM cells will outcompete CCR2-deficent cells in the reconstitution of CCR2 dependent cell types, resulting in the complete repopulation of LN monocytes by H–2K^bm1^-derived BM cells (or CD45.1 WT BM cells in control mice). Importantly DCs, which do not depend on CCR2 for their migration into tissue, are found at a ratio of 80:20 CD45.2:CD45.1 in the LNs of control mice, reflecting the ratio of transferred BM cells (**Figure 2A**). Thus, in chimeric mice, 100% of LN monocytes are incapable of directly presenting antigen to OTI T cells while antigen presentation by DCs is minimally affected.

In BM chimeras where the LN monocytes were derived from WT or H–2K^bm1^ BM, we observed no difference in the cytokine production in Poly I:C induced CTLs (**Figure 2C**). Therefore, we examined our second hypothesis that PDL1 expression on monocytes directly dampen the CTL response. In the LLN, PDL1 is highly expressed in steady-state and inflamed monocytes (**Figure 2B**). However, in the lung, monocytes and not DCs upregulate PDL1 after Poly I:C exposure (**Figure 2B**) (7). When we created BM chimeric mice where monocytes lacked PDL1/2 expression, there was a slight decrease in cytokine production from proliferating antigen-specific T cells compared to PDL1/2 expressing monocytes (**Figure 2D**). In summary, the mechanism of action that LN monocytes use to regulate a Poly I:C-induced antigen-specific CTL response is not mediated *via* cross-presentation or PDL1 expression.

**Figure 3:**
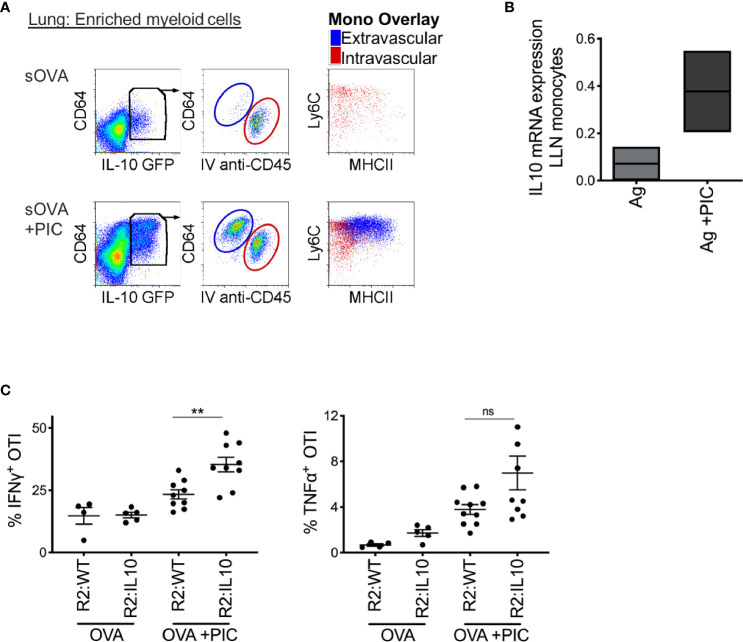
Poly IC-induced IL-10 production by monocytes is required to suppress antigen-specific CTL response. **(A)** IL-10 GFP reporter mice were inoculated i.n. with 2 μg OVA ±20 μg Poly IC. Five minutes prior to sacrifice and tissue harvest, 5 ul of anti-CD45 antibody was given intravenously. Lung monocytes were isolated and analyzed by flow cytometry for IL-10 expression, left plots. Middle plots illustrate gated IL-10 producing cells, where all extravascular IL-10 producing cells were CD64^+^ cells. Right plots display overlay of intravascular (red) and extravascular (blue) IL-10 producing cells, which were all CD64^+^ Ly6C^+^ monocytes with extravascular monocytes displaying signs of maturation, up-regulation of MHCII. **(B)** Normalized mRNA expression of IL-10 in LLN of WT mice after i.n. immunization with 2 μg OVA ±20 μg Poly IC as outlined in **Figure 1B**. Box plot is the mean of two independent experiments. **(C)** Lethally irradiated mice were reconstituted 80:20 with CCR2^-/-^ and IL-10^-/-^ BM cells: R2:IL-10. Control mice were reconstituted 80:20 with CCR2^-/-^ and WT BM cells: R2:WT chimeric mice. Scatter plot analysis of cytokine frequency from R2:WT and R2:IL-10 chimeric mice. Datum is representative of two independent experiments. Data are shown as mean ± SEM. Each dot represents a mouse. **P value < 0.01. Non-significant (ns).

Thirdly, we hypothesized that the production of IL-10, a well-described anti-inflammatory cytokine secreted by monocytes and macrophages in response to PAMP stimulation, might provide the mechanism by which monocytes dampen a Poly I:C-induced CTL response. First, we confirmed that monocytes produce IL-10 after an intranasal bolus of antigen and Poly I:C using IL-10 reporter mice (**Figure 3A**). Among extravascular CD11b^+^ cells, CD64^+^Ly6C^+^ monocytes uniquely expressed IL-10 after exposure to Poly I:C; intravascular cells were excluded by labeling with injection of anti-CD45 antibody given 5 minutes prior to harvest. Moreover, in the LLN, monocytes increased their mRNA expression of IL-10 after nasal inoculation of antigen and Poly I:C (**Figure 3B**). Using the same bone marrow chimera approach described previously (**Figure 2**), we created mice with IL-10 deficient LN monocytes. Compared to IL-10 sufficient LN monocytes, IL-10 deficient LN monocytes were unable to dampen the CTL response induced by antigen and Poly I:C (**Figure 3C**). In conclusion, these data suggest that monocytes require IL-10 production to dampen a CTL response. However, whether this effect is direct or indirect is unclear.

### Monocytes Are Required for the Induction of Suppressor Antigen-Specific T Cells

**Figure 4:**
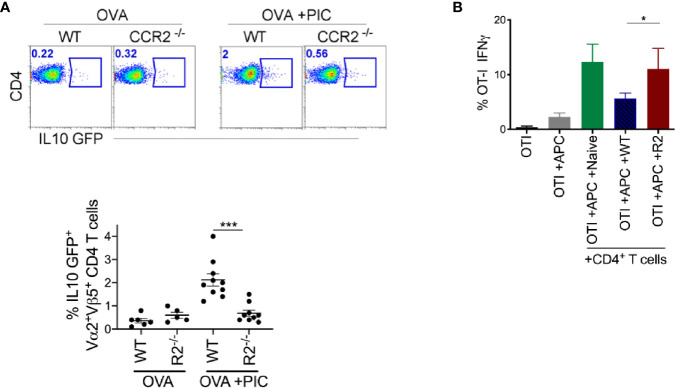
Monocytes are required for the induction of suppressive CD4^+^ T cells. **(A)** After immunization as outlined in **Figure 1B** and adoptive transfer of OTII IL-10 GFP^+^ cells, LLN antigen-specific OTII IL-10 GFP^+^ T cells was examined in WT and CCR2^-/-^ mice. Representative flow plots display OTII population gated on Vα2^+^Vβ5^+^CD4^+^IL-10 GFP^+^ from WT and CCR2^-/-^ mice, top. Scatter plot analysis of targeted population frequency from individual mice, Bottom. Combined set of 2 independent experiment. **(B)** WT splenic DCs and CFSE-labeled OTI cells were co-cultured with CD4^+^ T cells isolated from LLN of either naïve mice or WT and CCR2^-/-^ mice immunized with OVA ± Poly IC as outlined in **Figure 1B**. Bar graph demonstrates IFNγ (left) and TNFα (right) producing OTI T cells after 5 days of co-culture. Data are representative of two independent experiments, n=4 mice per group. Data are shown as mean ± SEM. ***P value < 0.001. *P < 0.05.

Next, we investigated whether other immune cell populations were involved in the modulation of adaptive immune response promoted by monocytes. Suppressor T cells (Treg) are known for their critical role in controlling autoimmunity and suppressing inflammatory responses. They also promote tumor development and progression by dampening the anti-tumor immune response. Therefore, we examined whether there was an increase in suppressor T cells in WT mice compared to CCR2^-/-^ mice. Suppressor T cells can be either Foxp3^+^ or Foxp3^-^ (35). We observed no difference in the frequency of Foxp3^+^ regulatory T cells between WT and CCR2^-/-^ mice (**Supplementary Figure 2**). Then, we examined the frequency of endogenous CD44^+^CD4^+^ IL-10 producing T cells post antigen and Poly I:C exposure and observed an increase in IL-10 producing CD4^+^ T cells in WT mice compared to CCR2^-/-^ mice (**Supplementary Figure 2**). Since these data do not convey antigen-specificity, OTII OVA-specific CD4 T cells were crossed with an IL-10 reporter and transferred these into WT or CCR2^-/-^ mice. After delivery of antigen with Poly I:C, WT mice displayed a significant increase in IL-10 producing antigen-specific CD4^+^ T cells compared to CCR2^-/-^ monocyte deficient mice (**Figure 4A**).

Based on this finding, we examined *ex vivo* whether endogenous CD4^+^ T cells from antigen and Poly I:C treated WT mice are more suppressive than those from CCR2^-/-^ mice. We isolated CD4^+^ T cells from the LLNs of WT and CCR2^-/-^ mice treated with antigen and Poly I:C and co-cultured them with antigen-specific CD8 T cells and stimulants (splenic DCs, antigen and Poly I:C). After five days in culture, antigen-specific CD8^+^ T cells co-cultured with CD4^+^ T cells from WT mice compared to CCR2^-/-^ mice produced significantly less IFNγ (**Figure 4B**). This suggests that LN monocytes are required for the induction of an antigen-specific CD4^+^ T cell that suppresses the Poly I:C induced CTL response.

**Figure 5:**
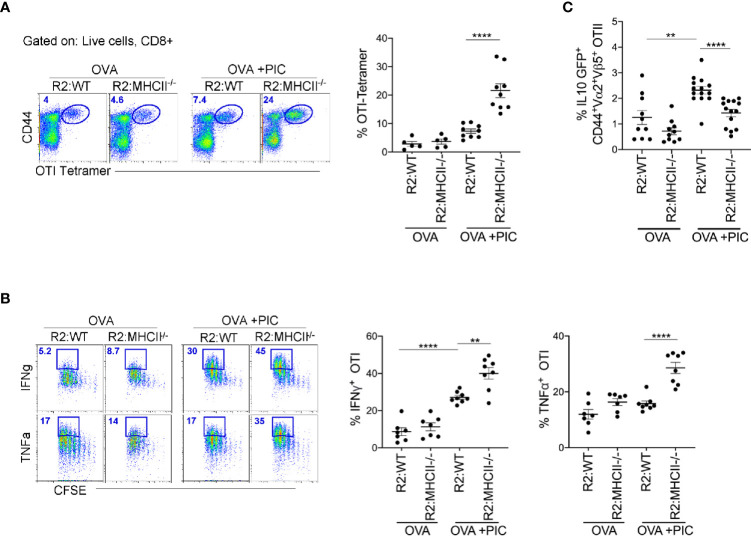
Poly IC-induced IL-10 production to suppress antigen-specific CTL response is done by MHCII^+^ monocytes. **(A)** OTI SIINFEKL tetramer^+^ T cells was examined in BM Chimeric mice after immunization and adoptive transfer (i.v.) of OTI cells. Scatter plot shows analysis of targeted population frequency from individual mice (right) and flow panel illustrate the representative CD44^+^ Tetramer^+^ OTI cells (left). Combined set of 2 independent experiment, n=3-5 mice per group. **(B)** After immunization as outlined in **Figure 1B**, LLN antigen-specific OTII IL-10 GFP^+^ T cells was examined in BM Chimeric mice. Combined set of 3 independent experiments, n=3-5 mice per group**. (C)** Lethally irradiated mice were reconstituted 80:20 with CCR2^-/-^ and MHCII^-/-^ BM cells: R2:MHCII chimeric mice. Control mice were reconstituted 80:20 with CCR2^-/-^ and WT BM cells: R2:WT chimeric mice. Flow and Scatter plot analysis of cytokine frequency from respective BM chimeric mice. Representative data of 2 independent experiments, n=3-5 mice per group. Data are shown as mean ± SEM. Each dot represents a mouse. ****P value < 0.0001. **P < 0.01.

To clearly demonstrate that antigen presentation by monocytes is required for the induction of suppressor T cells, we created BM chimeric mice where monocytes lacked MHCII expression (80:20, CCR2^-/-^MHCII^-/-^ BM). First, we examined whether endogenous Poly I:C-induced antigen-specific CD8^+^ T cells were significantly increased when monocytes lacked the ability to differentiate suppressor CD4 T cells. Indeed, when monocytes lacked MHCII expression, tetramer staining illustrated a significant increase in endogenous Poly I:C induced antigen-specific CD8^+^ T cells (**Figure 5A**). When CCR2^-/-^:MHCII^-/-^ BM chimeras were challenged with antigen, Poly I:C, and antigen-specific CD8^+^ T cells, as in **Figure 1**, mice lacking MHCII expression on monocytes, displayed significantly enhanced cytokine production in Poly I:C-induced antigen-specific T cells (**Figure 5B**). Finally, we demonstrated in MHCII sufficient monocyte BM chimeras that the differentiation of IL-10 producing antigen-specific CD4^+^ T cells was significantly greater than in MHCII deficient monocyte BM chimeras (**Figure 5C**). Overall, these data suggest that MHCII expression on monocytes and not DCs is required for the differentiation of IL-10 suppressor antigen-specific CD4^+^ T cells that regulate the Poly I:C induced CTL response.

### Blocking IL-10 Improves Prophylactic Treatment in Metastatic Melanoma Model

**Figure 6:**
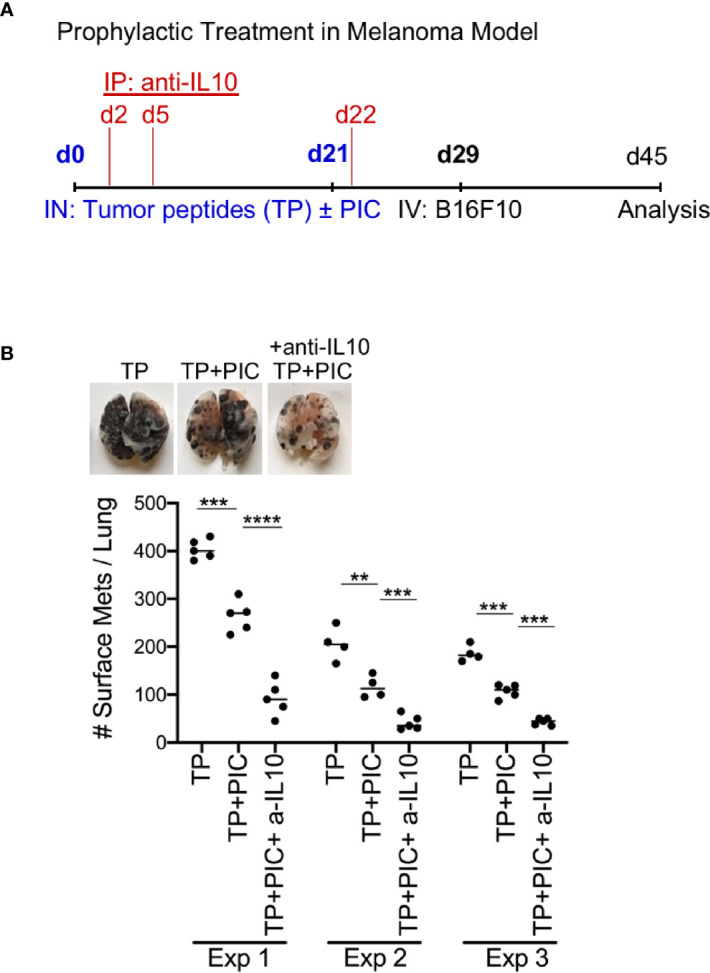
Immunization with B16F10 Peptides (TP) +Poly IC +anti IL-10 subunit vaccine produces anti-tumor effect in metastatic B16F10 melanoma model. **(A)** Schematic protocol for vaccination and B16F10 i.v. injection in WT mice for anti-tumor effect. **(B)** WT with TP (peptides) only, with TP +Poly IC and with TP +Poly IC +anti-IL-10 were harvested after i.v. B16F10 challenge and mouse lungs were inflated. Pics depict total surface metastases (mets) per lung (top), which were enumerated and illustrated by scatter plot, each dot represents one mouse (bottom). Combined data of three independent experiments with 4-5 mice per group. Data are shown as mean ± SEM. ****P value < 0.0001. ***P < 0.001. **P < 0.01.

Next, the relevance of these findings was demonstrated in a prophylactic metastatic melanoma model. Treatment with anti-IL-10 was performed instead of monocyte depletion, as there are currently no human monocyte specific depleting antibodies. A triple combination therapy of anti-IL-10, tumor peptides and Poly I:C, was compared to tumor peptides +/- Poly I:C, types of therapy already known to promote anti-tumor immunity. Although the tumor peptides with Poly I:C alone displayed a robust anti-tumor immune response, the triple combination therapy of melanoma challenged mice displayed the greatest anti-tumor immunity (**Figure 6** and **Supplementary Figure 3**). This finding demonstrates that an anti-tumor immune response can be significantly enhanced in combination with IL-10 neutralization.

### Human Lung-Draining LN Monocytes Selectively Express IL-10

**Figure 7:**
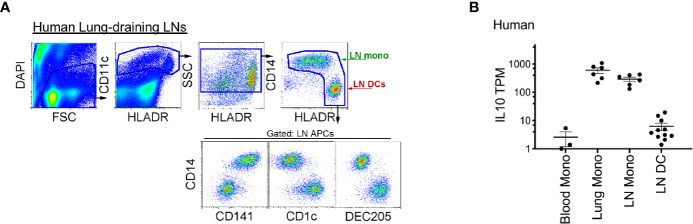
Compared to LN DCs, human lung and lung-draining LN monocytes express IL-10. **(A)** Top, flow cytometry gating strategy to identify human lung-draining LN monocytes and DCs: live cell gate, DAPI^-^, followed by CD11c^+^ cells and intermediate-high side scatter with the final HLADR^+/high^ APC gate: displaying CD14^+^CD141^low/+^ monocytes and DC subsets (CD14^-^CD141^-^, CD1c^+^, DEC205^+^). Flow plot is representative of over 30 human LLNs analyzed (36, 40). **(B)** RNA sequencing, transcripts per million (TPM) of monocytes and DCs sorted from human blood, lung and lung-draining LN (40). Data are shown as mean ± SEM.

Finally, to translate our findings to clinical practice, we examined whether human LN monocytes, like mice, express IL-10. Indeed, like mice, human LLNs contained a similar number of CD14^+^CD11c^hi^HLADR^+^ monocytes as CD14^-^CD11c^hi^HLADR^++^ DCs (**Figure 7A**) (36, 40), and expressed significantly more IL-10. Transcriptionally, the lung and LLN monocytes expressed ~10 to 100-fold greater amounts of IL-10 compared to LN DCs and immature blood monocytes (**Figure 7B**). This demonstrates that human monocytes produce IL-10 after extravasation into tissue and LN and perhaps, play a continuous role of regulating immunity.

## Discussion

In this study, we found that monocytes regulate Poly I:C-induced antigen-specific CTL response. First, we demonstrated that in the absence of LN monocytes (in CCR2^-/-^ mice), there was an enhanced Poly I:C-induced CTL response compared to WT mice. Second, using BM chimeric mice that lack key functional properties of monocytes—MHCI antigen cross-presentation, PDL1, IL-10, and MHCII antigen presentation—we found that the absence of IL-10 production and MHCII expression impaired monocyte function, resulting in enhanced CTL response. Third, we demonstrated that the production of IL-10 by monocytes was required to promote the differentiation of suppressor CD4^+^ T cells. When CD4^+^ T cells were isolated from WT mice inoculated with antigen and Poly I:C, and co-cultured with stimulated OTI T cells, CTL cytokines were significantly suppressed compared to CD4^+^ T cells isolated from monocyte deficient mice. These results demonstrate that the presence of monocytes is required to develop antigen-specific suppressor CD4^+^ T cells. Fourth, in a metastatic melanoma model, we examined the importance of removing the monocytic brake in a prophylactic treatment consisting of anti-IL-10, tumor peptides and Poly I:C. The triple combination therapy resulted in significantly fewer tumors compared to mice treated with tumor peptides and Poly I:C alone. Finally, we found that like mice, only human LN monocytes (but not DCs) expressed IL-10. This finding converges with other studies of human orthologs of splenic APCs, which show that monocytes (compared to other cell types) have strong transcriptional correspondence across species (1, 41). Overall, our findings suggest that monocytes exert a braking function on Poly I:C-induced CTL responses through the production of IL-10 and indirectly *via* the induction of suppressor CD4+ T cells. This novel function of LN monocytes limits the potential for DC-mediated over-exuberant CTL responses that might be damaging to the host. The development of a suppressor CD4+ T cell population is likely important in returning the immune system to homeostasis after an inflammatory response.

Our findings have implications for the development of vaccines and other therapeutics that depend on Poly I:C. The future of personalized medicine is here, and the synthesis of personalized neoantigens with Poly I:C and immune checkpoint blockade is currently in clinical trials. Although these trials appear to be efficacious, understanding what may potentiate or limit the efficiency is critical in developing additional treatments for combination therapy, such as IL-10 as demonstrated here. In addition, the tumor microenvironment contains suppressive monocyte-derived cells which can be targeted in a similar fashion as illustrated here for LN monocytes. Thus, defining the role each antigen-presenting cell subtype may play during the induction of the desired cancer or pathogen vaccine is imperative and may enhance our ability to develop immunotherapies for cancer and other diseases.

## Data Availability Statement

The raw data supporting the conclusions of this article will be made available by the authors, without undue reservation.

## Ethics Statement

The animal study was reviewed and approved by National Jewish Health and Dartmouth Hitchcock Medical College, an AAALAC accredited institution, and used in accordance with protocols approved by the Institutional Animal Care and Utilization Committee.

## Author Contributions

AT, MP, SG, and CJ contributed to concept, experimental design, and wrote the manuscript. AT, MP, SG, and KR performed the animal experiments and analyzed the data. All authors contributed to the article and approved the submitted version.

## Funding

National Institutes of Health supported this work CJ grants: R01 HL115334, R01 HL135001 and R35 HL155458 (CJ).

## Conflict of Interest

The authors declare that the research was conducted in the absence of any commercial or financial relationships that could be construed as a potential conflict of interest.

## Publisher’s Note

All claims expressed in this article are solely those of the authors and do not necessarily represent those of their affiliated organizations, or those of the publisher, the editors and the reviewers. Any product that may be evaluated in this article, or claim that may be made by its manufacturer, is not guaranteed or endorsed by the publisher.
