# Supplementary material for: LN Monocytes Limit DC-Poly I:C Induced Cytotoxic T Cell Response via IL-10 and Induction of Suppressor CD4 T Cells
Source: Front Immunol. 2021 Oct 6;12:763379. doi: 10.3389/fimmu.2021.763379 (PMC8527167; doi:10.3389/fimmu.2021.763379)
Supplement: Supplementary file 1 [file DataSheet_1.pdf]

Supplementary Figures

LN monocytes limit DC-Poly I:C induced cytotoxic T cell response via IL-10 and induction of suppressor CD4 T cells

Anita Tewari[1*], Miglena G. Prabagar[2*], Sophie L. Gibbings[2*], Kavita Rawat[1], and Claudia V. Jakubzick[1]

[1]Department of Microbiology and Immunology, Geisel School of Medicine, Hanover, NH, USA 03756, [2]Department of Pediatrics, National Jewish Health, Denver, CO, USA 80206
*equal contribution

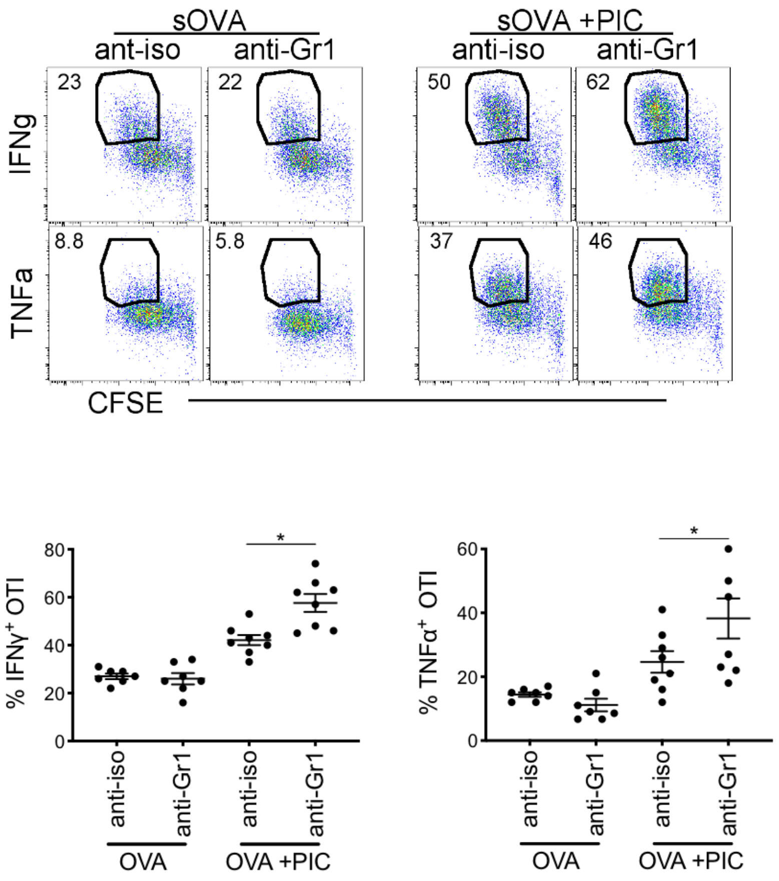

Supplementary Figure 1. Transient depletion of monocytes with anti-Gr1 antibody enhanced Poly IC-induced CTL response

WT mice were given 300 μg of anti-Gr1 or isotype control on the same day as CFSE-labeled OTI T cells. Three days post immunization as illustrated in Figure 1B, LLNs were assessed for cytokine frequency in proliferating OTI cells. Top, representative flow plots display gating strategy of cytokine production from WT isotype and anti-Gr1 treated mice. Bottom, scatter plot analysis of cytokine frequency from individual mice. Data are cumulative from 2 independent experiments of n=3,4 per group. Each dot represents a mouse. *P value < 0.05.

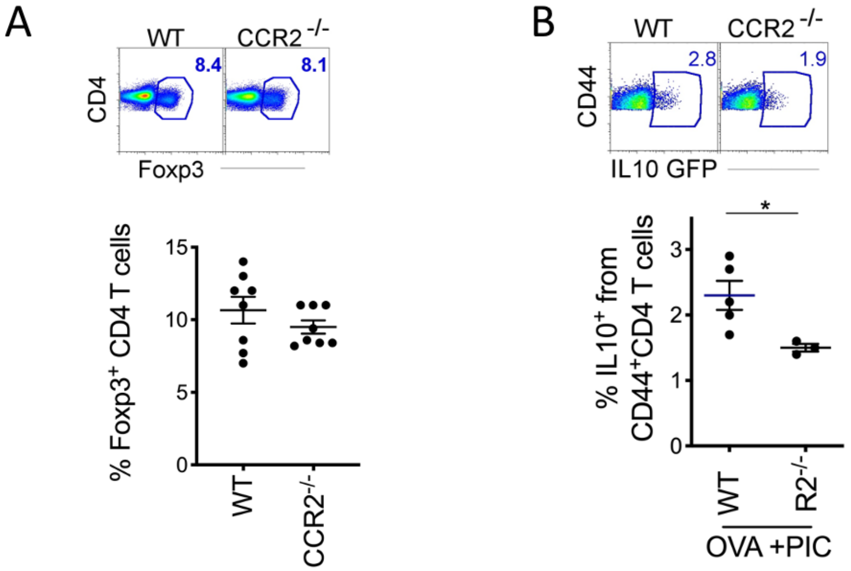

Supplementary Figure 2. **Monocytes are required for the induction of suppressive $CD4^+$ T cells.**

(A) Flow plots (top panel) and scatter plot analysis (bottom panel) illustrate frequency of $Foxp3^+$ CD4 T cells was examined in WT and $CCR2^{-/-}$ mice. **(B)** After immunization as outlined in Figure 1B, LLN antigen-specific IL-10 GFP producing $CD4^+$ T cells was examined in WT and $CCR2^{-/-}$ mice. Representative flow plots display gating strategy of IL-10 GFP producing $CD44^+CD4^+$ T cells from WT and $CCR2^{-/-}$ mice, top. Scatter plot analysis of targeted population frequency from individual mice, bottom. Data are shown as mean ± SEM. *P value < 0.05.

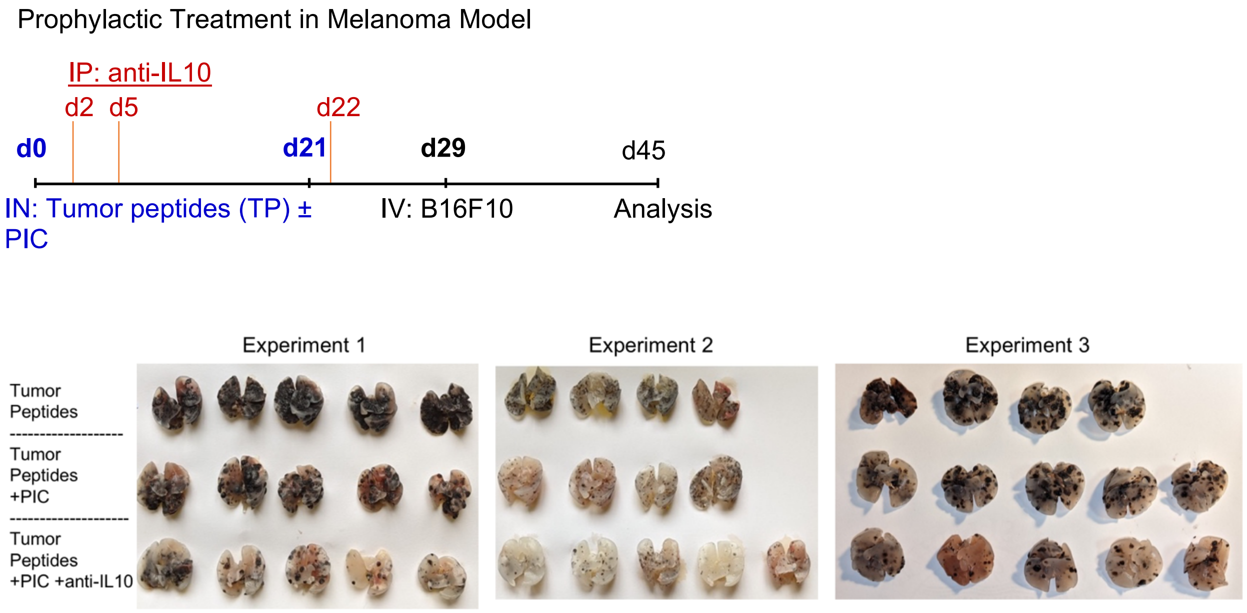

Supplementary Figure 3. Immunization with Peptide (TP) +Poly I:C +anti-IL-10 subunit vaccine produces anti-tumor effect in metastatic B16F10 melanoma model WT mice lungs with TP (Tumor peptide) only, with TP + Poly I:C or with TP +Poly I:C +anti-IL-10 were harvested after i.v. B16F10 challenge and mouse lungs were inflated. Pics depict total surface metastases (mets) per lung. Presented data is from three independent experiments.
